# Geochemical insights and model optimisation for pilot-scale passive treatment of manganese and zinc in a legacy mine in Japan

**DOI:** 10.1016/j.heliyon.2024.e40363

**Published:** 2024-11-13

**Authors:** Sereyroith Tum, Taiki Katayama, Naoyuki Miyata, Miho Watanabe, Yohey Hashimoto, Miu Nishikata, Tetsuo Yasutaka

**Affiliations:** a Institute for Geo-Resources and Environment, Geological Survey of Japan, National Institute of Advanced Industrial Science and Technology (AIST), 1-1-1 Central 7, Higashi, 305-8567, Tsukuba, Ibaraki, Japan; b Department of Biological Environment, Akita Prefectural University, 241-438, Shimoshinjo-Nakano, 010-0195, Akita, Japan; c Department of Bioapplications and Systems Engineering (BASE), Tokyo University of Agriculture and Technology, 2-24-16, Nakamachi Koganei,184-8588, Tokyo, Japan

**Keywords:** Mn-oxidising bacteria, Mn^2+^ oxidation rate constant, Mass transfer, Distribution coefficient, Hydraulic retention time

## Abstract

Elevated concentrations of manganese (Mn^2+^) and zinc (Zn^2+^) in water bodies can disrupt ecosystems and damage aquatic life. However, the mechanisms underlying the removal of Mn^2+^ and Zn^2+^ under dynamic conditions and the optimal hydraulic retention time (HRT) for passive treatment plants remain unclear. Here, a pilot-scale passive treatment system for the removal of Mn^2+^ and Zn^2+^ from legacy mine drainage in northern Japan is proposed; it was performed at circumneutral pH for 152 days. Comprehensive suspended solid mineralogy analyses and geochemical and numerical modelling were conducted to optimise the passive treatment efficiency. Mn^2+^ removal (efficiency reaching 98 %) primarily depended on the activity of Mn-oxidising bacteria. Zn^2+^ removal involved Zn^2+^ co-precipitation with birnessite combined with adsorption or ion exchange on the birnessite surface. The inverse numerical model successfully determined the Mn^2+^ oxidation rate constant, Zn mass transfer coefficient, and Zn distribution coefficient. Under dynamic conditions, HRT emerged as a key factor underlying the pilot-scale passive treatment efficiency. An HRT of 0.5 days led to optimal Mn^2+^ and Zn^2+^ removal conditions and achieved values lower than the Japanese national effluent limit. The findings provide crucial information for passive treatment strategy development and environmental management, especially when considering real-scale implementation.

## Highlights

• Passive treatment is sustainable for treating mine drainage in legacy mines.
• Passive treatment is applicable when Mn, Mn-oxidising bacteria, and Zn coexist.
• Pilot-scale passive treatment achieved high-efficiency removal of Mn and Zn.
• Inverse modeling is favorable for determining geochemical parameters.
• Hydraulic retention time influences Mn^2+^ and Zn^2+^ treatment efficiency.

## Graphical abstract

**Figure:**
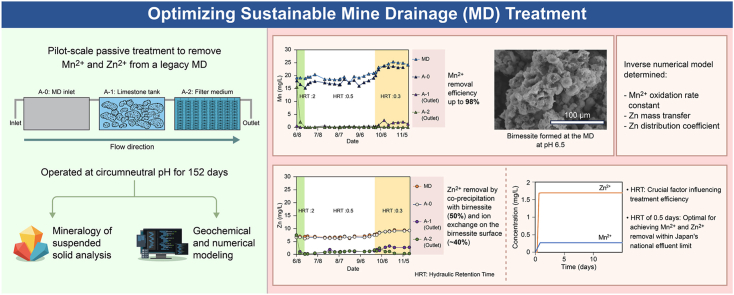

**Environmental implication:** Dissolved Mn^2+^ and Zn^2+^ are hazardous elements that significantly affect human health and aquatic ecosystems. This study presents an innovative approach for the sustainable treatment of Mn^2+^ and Zn^2+^ using passive treatment methods. Understanding the mechanisms underlying Mn^2+^ and Zn^2+^ removal can enable researchers to design suitable passive treatment strategies. Additionally, the application of inverse modelling techniques to monitoring data reduces the cost and time associated with traditional sorption experiments. These solutions contribute to environmentally friendly and economically viable approaches for Mn^2+^ and Zn^2+^ treatment, thereby addressing the critical need for sustainable and effective solutions.

## 1 Introduction

Mine drainage is a significant environmental concern because it releases an array of highly toxic metals and metalloids into aquatic ecosystems [[1], [2], [3]]. Among these contaminants, dissolved manganese (Mn^2+^) and zinc (Zn^2+^) are particularly alarming because of their pervasive nature and harmful environmental consequences. Elevated concentrations of Mn^2+^ and Zn^2+^ in water bodies can disrupt ecosystems and damage aquatic life and pose potential health hazards to humans [4,5]. Consequently, the development of efficient and sustainable methods for the removal of Mn^2+^ and Zn^2+^ during mine drainage treatment is necessary. Passive treatment systems have emerged as cost-effective solutions for treating legacy mine sites [6,7]. Although the efficacy of passive treatment has been demonstrated, the implementation of such systems is not universally feasible because of variations in physical and geochemical characteristics across mining sites [6]. These disparities underscore the pressing need for innovative solutions, particularly for Mn^2+^ and Zn^2+^ treatment of mine drainage material. Various techniques have been used for Mn^2+^ removal, including oxidation and precipitation processes [8]. Mn^2+^ undergoes transformation into the species Mn^3+^, Mn^4+^, and Mn^7+^ [9], and it eventually precipitates as minerals, such as manganese oxides, todorokite, pyrolusite, birnessite, manganite, and manganese hydroxide [2,10,11]. Under abiotic conditions, Mn^2+^ oxidation requires a high pH (>10) [8], whereas under biotic conditions, Mn^2+^ oxidation can occur at circumneutral pH levels [[11], [12], [13]]. Conversely, Zn^2+^ removal relies predominantly on geochemical conditions rather than biological processes. Dissolved Zn^2+^ has been reported to incorporate manganese oxides through processes such as ion exchange, adsorption, and co-precipitation, particularly at pH levels of 6.5–7.5 [[14], [15], [16]]. The inherent association between Zn^2+^ and manganese oxides presents an intriguing opportunity to leverage Mn-oxidising bacteria (MnOB) in passive biological treatment systems.

A recent study showed that the MnOB microbial community serves as a Mn^2+^ oxidation catalyst for removing Mn^2+^ and Zn^2+^ from mine drainage under organic substrate-limited conditions [17]. Although Mn^2+^ and Zn^2+^ have been successfully removed from mine drainage, previous studies have focused extensively on the role of bacterial species in the treatment system. Thus, gaps remain in our understanding of the mechanisms underlying the removal of Mn^2+^ and Zn^2+^ under dynamic conditions and the optimal hydraulic retention time (HRT) for passive treatment plants.

Therefore, the objectives of this study were to: (1) characterise the treatment mechanisms for Mn^2+^ and Zn^2+^ in a pilot-scale passive treatment and (2) develop a numerical kinetic model for the removal of Mn^2+^ and Zn^2+^ based on treatment mechanism analyses and field measurements. To determine the treatment mechanisms of Mn^2+^ and Zn^2+^, suspended solids in the mine drainage and treatment tanks were subjected to detailed mineralogical analyses, which were supported by geochemical modelling. An inverse numerical model of the pilot-scale passive treatment field experiments was reconstructed to determine the Mn^2+^ oxidation rate constant, Zn^2+^ mass transfer coefficient, and Zn^2+^distribution coefficient with manganese oxide. Our study revealed the mechanisms underlying the pilot-scale treatment process and showed that field monitoring data can be used to obtain geochemical parameters, and numerical modelling can be used to optimise model parameters.

## 2 Material and methods

### 2.1 Pilot-scale passive treatment

A pilot-scale passive treatment was conducted at a legacy mine X in northern Japan, which is a hydrothermal vein deposit rich in sphalerite (PbS), pyrite (FeS), and rhodochrosite (MnCO_3_) [18,19]. Although mine activity was terminated in 1979, active treatment methods continue to be applied to the mine drainage.

**Fig. 1:**
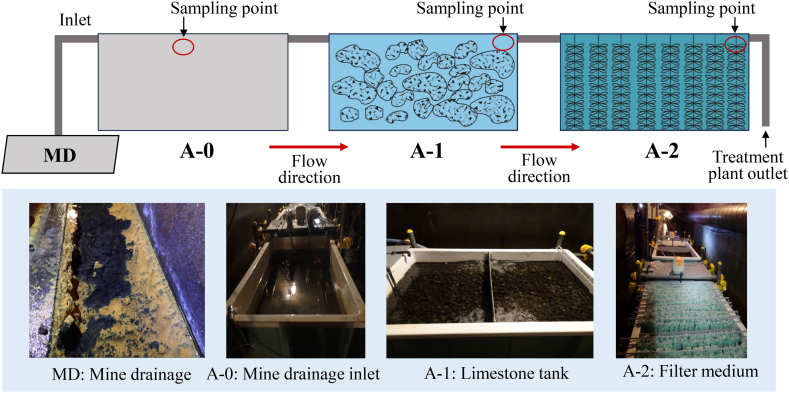
Schematic of the pilot-scale passive treatment plant operated inside the underground tunnel of the legacy mine X. Sampling points A-1 and A-2 were located at the outlet of the tank.

The field investigation and data collection in this study were performed during a second pilot-scale experiment conducted over 152 days from June to November 2022. The pilot-scale passive treatment system was implemented with a circumneutral pH range of 6.5–7.5 under oxic conditions. As shown in Fig. 1, the mine drainage from a small stream (MD) at mine X was pumped into A-0, which was then fed into A-1, a passive treatment tank filled with limestone. The treated water from A-1 flowed into A-2, another passive treatment tank equipped with a fibre filter to increase the effective volume of the bioreactors. Each of these tanks (A-0, A-1, and A-2) had a volume of 600 L. Notably, A-1 had a porosity of 48 %, whereas A-2 had a porosity of 92 %. Air agitators were installed at A-1 and A-2 as oxygen suppliers.

The first experiment was performed during the summer of 2021 and lasted approximately 150 days [20]; however, the second experiment was postponed due to inaccessibility during the winter months. During the first experiment, approximately 2.5 L of suspended solids from the MD was used as the initial source of MnOB [20] in A-1, while 1 L was used in A-2. However, when the second experiment was performed, the limestone in A-1 and fibre filter in A-2 were entirely coated with black suspended solids. The total HRT from the inlet of A-1 to the outlet of A-2 was continuously operated with three different HRTs; 6 days over 16 days, 1.5 days over 97 days, and 1 day over 37 days. The HRT in each tank set was determined separately because of differences in the porosity of each tank. In A-1, the HRT was set to 2, 0.5, and 0.3 days, whereas in A-2, it was set to 4, 1, and 0.7 days.

### 2.2 Analytical methods

Water samples were collected periodically in 2022 from the MD, A-0, A-1, and A-2 and sent to Akita Prefectural University for Mn and Zn analyses by inductively coupled plasma-optical emission spectroscopy (ICP-OES; iCAP 6000, Thermo Fisher Scientific, San Jose, USA). During water sampling, the electrical conductivity (EC), dissolved oxygen (DO), oxidation-reduction potential (ORP), and pH were measured at the study site. Solid samples, including naturally suspended solids from the small stream (MD) and suspended solids from treatments A-1 and A-2, were collected in September 2022 for mineralogical and chemical analyses. For simplicity, the suspended solid samples from A-1 and A-2 are referred to as ‘sludge’.

The mineralogy of the suspended solids was determined using X-ray diffraction (XRD, Rigaku RINT-2500, GSJ-Lab, AIST, Japan), with the working voltage 40 kV at 100 mA, 2Ɵ(°) CuKɑ range from 5° to 70°, the scanning speed 0.5° per min. The XRD pattern of the mineral was identified using Match software (Crystal Impact). The XRD pattern of woodruffite was compared with the data on Joint Committee on Powder Diffraction card number 47–1825 (International Centre for Diffraction Data). The morphologies of the solid samples were characterised using scanning electron microscopy with energy-dispersive X-ray spectroscopy (SEM-EDS, SU3500, HITACHI, GSJ-Lab, AIST, Japan). The powdered samples were freeze-dried, attached to silicon glass beads, and coated with osmium for SEM-EDS analysis. The SEM-EDS accelerating voltage was 15 kV, and the displayed signal was scattered electrons (SEs). The chemical composition of the solid samples was determined using X-ray fluorescence (XRF; Rigaku Supermini200, Japan).

### 2.3 X-ray absorption fine structure spectroscopy analysis

X-ray absorption fine structure spectroscopy (XAFS) analyses included both extended X-ray absorption fine structure (EXAFS) and X-ray absorption near edge structure (XANES) analyses, which were performed using beamline BL5S1 at the Aichi Synchrotron Radiation Centre, Aichi, Japan. The dry solid samples were diluted to ∼1 % with boron nitride (BN) powder [21] targeted for Mn K-edge and Zn K-edge XAFS spectra separately and then pressed into pellets (10 mm diameter and 1 mm thickness).

Mn K-edge XAS spectra of the solid samples were obtained from 6392 to 7316 eV in transmission mode using a Si (111) monochromator crystal at ambient temperature, and the measurement energy was calibrated by the white line peak of Mn foil (MN00-FL-00190, Goodfellow, England) at E_0_ = 6546 eV. Standard compounds for the Mn K-edge and Zn K-edge XAFS measurements included MnCO_3_, MnO (Mn^II^), Mn_2_O_3_ (Mn^III^), Mn_3_O_4_ (Mn^II,III^), and birnessite (MnO_2_, Mn^IV^), and they were synthesised in the laboratory according to the method described by Tajima et al. [16].

Zn K-edge XAS spectra of the solid samples were obtained in the range of 9363–10163 eV in transmission mode using a silica (111) monochromator crystal at ambient temperature, and the measurement energy was calibrated using the white line peak of Zn foil (ZN00-FL-000110, Goodfellow, England) at E_0_ = 9663 eV. The reference material sample Zn compounds were ZnO, ZnCO_3_, ZnS, adsorption Zn^2+^ on birnessite (Zn_ads), and Zn^2+^ coprecipitation with birnessite (Zn_co). Standard samples for adsorption Zn^2+^ on birnessite and Zn^2+^ coprecipitation with birnessite were synthesised based on the method described by Tajima et al. [16].

XAFS analytical data from the solid samples were processed using the Athena XAS data-processing software package (version 0.9.26) for data calibration and interpretation. The oxidation states and quantitative mineral compositions of the samples were determined using Mn and Zn XANES spectra. The local structures of Mn and Zn minerals were determined based on the normalised k^2^χ(k) (A^−2^) of EXAFS spectra between wavelengths of 2–10 Å^−1^ and Fourier transforms of normalised χ(R) (A^−4^) of EXAFS spectra with radial distances (R+△R) from 0 to 6 Å. Linear combination fitting (LCF) was performed on the normalised XANES spectra samples within a relative energy fitting range between −20 and 30 eV using the Athena software package.

### 2.4 Three-step sequential extraction

Sequential extraction of suspended solids from the MD and sludge was performed using the three-step Community Bureau of Reference (BCR) method, as described by Pueyo et al. [22]. This three-step sequential extraction uses 0.11 mol/L acetic acid for fraction F1, 0.5 mol/L hydroxylammonium chloride for fraction F2, and 8.8 mol/L hydrogen peroxide and 1 mol/L ammonium acetate, with the pH adjusted to 2 using HNO_3_ for fraction F3 (Table 1). The extraction procedure for each fraction (F1, F2, and F3) is described by Pueyo et al. [22]. Aqueous samples from each fraction were sent to a commercial company for the analysis of elements, such as total Mn, Zn, Fe, and Ca.

**Table 1:** Description of sequential extraction fractionation using BCR method by Pueyo et al. [22].

Fraction	Solutions	Targets
F1	Acetic acid (0.11 mol/L)	Extracts the water, acid-soluble or ion exchangeable metals
F2	Hydroxylammonium chloride (0.5 mol/L)	Iron/manganese oxides minerals phase
F3	Hydrogen peroxide (8.8 mol/L, 30 %), D: Ammonium acetate (1 mol/L), adjusting the pH to 2 with HNO_3_.	Organic matter or the oxidisable minerals.

### 2.5 Geochemical and numerical modelling

The mineral saturation index (S.I.) was calculated using the Geochemist's Spreadsheet (GSS) module of the Geochemist's Workbench (GWB) version 17.0 professional package. The Eh-pH diagram of the Mn and Zn species was obtained from the Act2 module of GWB using the thermo.dat database. The one-dimension (1D) transport model was employed along with the kinetic reaction model via PHREEQC computer code and the wateq4f thermodynamic database [23]. A 1D path length of 1.6 m represents the A-1 water tank. The flow rate was calculated based on the HRT. The descriptive statistic the water quality parameters was obtained by R programming (R version 4.2.0). The input transport parameters, aqueous model, and initial and boundary conditions are presented in supplementary S1. An inverse model of the Mn oxidation kinetics and Zn sorption kinetic transport was constructed by coupling it with parameter estimation (PEST version 17.4) computer codes [24] to determine unknown parameters including; Mn oxidation rate constant (k_1_), Zn mass transfer coefficient (k_m_), and Zn distribution coefficient (K_d_). The Mn^2+^ and Zn^2+^ concentrations and water quality in A-0 represent the input of the flushing solution in the model, while the A-1 water quality represents the result of the reaction transport in the passive treatment plan. Three calibration datasets from A-1 were used to obtain the best fit for the inverse model. (1) The total Mn and Zn concentrations derived from the monitoring data in 2021 [20] were used as the calibration dataset (trained dataset), and the monitoring data in 2022 were used as the validation dataset. (2)The total Mn and Zn concentrations derived from the monitoring data in 2022 with an HRT of 0.5 days and monitoring data in 2022 with an HRT of 0.3 days were used as the validation dataset. (3)The total Mn and Zn concentrations derived from the monitoring data in 2022 with an HRT of 0.3 days and monitoring data in 2022 with an HRT of 0.5 days were used as the validation dataset (Supplementary S1). A gradient-based algorithm in PEST was used to minimise the weighted least-squares objective function, as described by Knowling and Werner [25]. The mean absolute error (MAE), root mean square error (RMSE), normalised root mean square error (NRMSE), and Goodness-of-fit or coefficient of efficiency R-square (R^2^) (Supplementary S1) were used to calculate error Mn and Zn concentration predicted by the reaction transport model to the monitoring data measured at the field [[26], [27], [28]]. The obtained parameter with the lowest value of NRMSE is taken for the future prediction of optimum HRT in for the passive treatment plan.

## 3 Results and discussion

### 3.1 Mineralogy of suspended solid and sludge

**Fig. 2:**
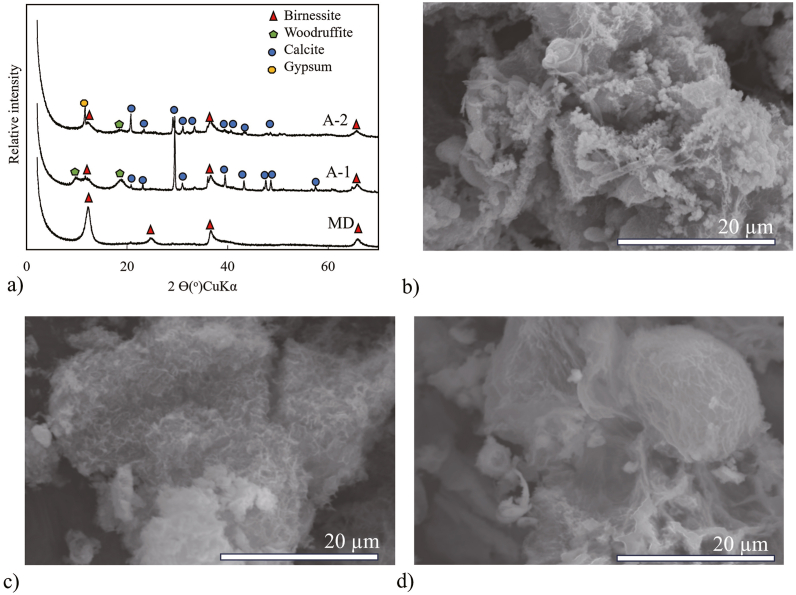
a) X-diffraction (XRD) patterns of the mineral drainage (MD) and sludge sample in A-1 and A-2; b) scanning electron microscopy (SEM) image of the suspended solid from MD showing the dissemination of iron oxyhydroxide twisted stalks organo-mineral on birnessite and spherical to elongate shape with fibrous nanostructure of birnessite; c) SEM image of sludge in A-1 showing that birnessite has an irregular shape and fibrous nanostructure; d) SEM image of sludge in A-2 showing that birnessite had a spherical shape and fibrous nanostructure.

The black suspended solid collected from the MD was identified as birnessite (MnO_2_) according to the XRD pattern, as shown in Fig. 2a. In addition, a yellowish suspended mineral (Fig. 1) precipitated above the birnessite, although it did not exhibit a distinct presence in the XRD pattern. It is believed to have a Fe oxyhydroxide twisted stalk organo-mineral structure that presents as a spiral-like structure disseminated on the spherical birnessite (Fig. 2b) resulting from the activity of Fe-oxidising bacteria [29]. Fe- and Mn-oxidising bacteria are recognised for their catalytic potential in the formation of iron hydroxide and manganese oxide minerals [30].

The sludge that precipitated in A-1 and A-2 was a mixture of birnessite, woodruffite, and calcite (Fig. 2a), although A-2 also contained gypsum and had a low-calcite XRD pattern. The yellowish Fe oxyhydroxide twisted stalk organo-mineral could not be seen by the naked eye in the treatment plant, although it was observed in A-1 and A-2 when analysed by SEM. Woodruffite, calcite, and gypsum were the most recent minerals formed in samples A-1 and A-2 when limestone was added to the pilot-scale passive treatment. The birnessite structure in the A-1 precipitates was irregular in shape, with fibrous nanostructures mixed with calcite (Fig. 2c). In addition, the birnessite in A-2 had a spherical shape and fibrous nanostructure (Fig. 2d) and was similar to the birnessite found in previous studies [31,32].

### 3.2 Water chemistry in the pilot-scale passive treatment

The pilot-scale passive treatment system was operated within a pH range of 6.5–7.5, with Mn^2+^/Zn^2+^ mole ratios of 2.5 in the MD. The MD flows through an underground mine tunnel and exhibits the following characteristics: average pH of 6.72, EC of 1138 μS/cm, DO at 5.02 mg/L, ORP of 138 mV, and average temperature of 15 °C during the summer (Supplementary S2). The neutral conditions of the MD allowed birnessite and other minerals to precipitate along a small channel, although the Mn^2+^ concentration remained at an almost constant value (∼20 mg/L). After the water from the MD was pumped into A-0, the pH and DO levels increased slightly to 6.87 and 7.73 mg/L, respectively, which was likely because of the higher water flow rate during pumping. In addition, the pH in A-1 gradually increased to 7.12, and as the MD flowed over the limestone gravel, the pH in A-2 slightly decreased to 7.04. Although large differences in pH, ORP, and EC were not observed between the MD and water in the pilot treatments (A-1 and A-2), notable changes were observed in DO, which reached approximately 9.3 mg/L in A-1 and A-2 owing to the use of artificial air agitation.

**Fig. 3:**
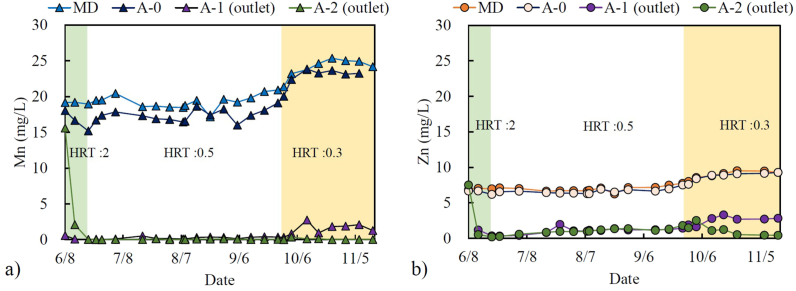
Monitoring data of the Mn and Zn concentrations from the pilot scale passive treatment from June to November 2022. a) Mn concentrations in the MD, A-0, A-1, and A-2; b) Zn concentrations in the MD, A-0, A-1, and A-2. The unit of hydraulic retention time (HRT) is days.

The concentrations of Mn^2+^ and Zn^2+^ were monitored over a 152-day period in the pilot-scale passive treatment, which employed various HRTs, as shown in Fig. 3. The Mn^2+^ concentration in the MD remained stable, with an average concentration of 19 mg/L from June to September 2022, but gradually increased to approximately 25 mg/L from October 2022. Similarly, the Zn^2+^ concentration in the MD was approximately 8 mg/L from June to September but increased to approximately 9.5 mg/L in October 2022. The increase in Mn^2+^ and Zn^2+^ concentrations in the MD may be attributed to seasonal variations, such as snow melting, summer rain, and water temperature, which affect MD water quality [[33], [34], [35]].

The A-0 water tank primarily served as a water storage tank, and it showed a slight decrease in Mn^2+^ concentration compared to that of the MD (approximately 2 mg/L) and slight precipitation of birnessite. The Mn^2+^ concentrations in A-1 and A-2 drastically decreased from 19 mg/L (A-0) to less than 0.5 mg/L, even when the HRT was changed from 2 to 0.5 days. However, a significant increase in Mn^2+^ concentration to over 2 mg/L was observed in A-1 when the HRT was reduced to 0.3 days (Fig. 3a). The Mn^2+^ concentration remained lower than the Japan national effluent limit (Mn = 10 mg/L) [36] after changing the HRT to 0.3 days, and the residual Mn^2+^ was further removed in A-2.

A similar trend was observed for the Zn^2+^ concentration in the treatment plant, which was slightly lower in A-0 than in the MD. The Zn^2+^ concentration remained steady at approximately 8 mg/L but showed a slight increase to approximately 9 mg/L in October (Fig. 3b). The Zn^2+^ treatment efficiency was not as high as that of Mn^2+^. The Zn^2+^ concentration significantly changed as the HRT changed. The average Zn^2+^ concentrations were 0.56, 1.59, and 2.79 mg/L for HRTs of 2, 0.5, and 0.3 days in A-1, respectively. Zn^2+^ was above the effluent limit (2 mg/L) when the passive treatment was operated at an HRT 0.3 days, although the residual Zn^2+^ was later removed in A-2.

A-1 and A-2 exhibited significant Mn^2+^ removal efficiency, with removal rates of 98 % at HRTs of 2 and 0.5 days; however, this rate decreased to 91 % for an HRT of 0.3 days. The rapid removal of Mn^2+^ within a short period resulted in sludge precipitation, with birnessite as the dominant mineral on the limestone gravel. In contrast, Zn^2+^ displayed different behaviours, with removal rates of 94 %, 82 %, and 69 % at HRTs of 2, 0.5, and 0.3 days, respectively. The Zn^2+^ removal efficiency decreased as the HRT in the treatment plant decreased.

### 3.3 X-ray absorption spectroscopy

#### 3.3.1 XANES and EXAFS analyses of Mn compounds

**Fig. 4:**
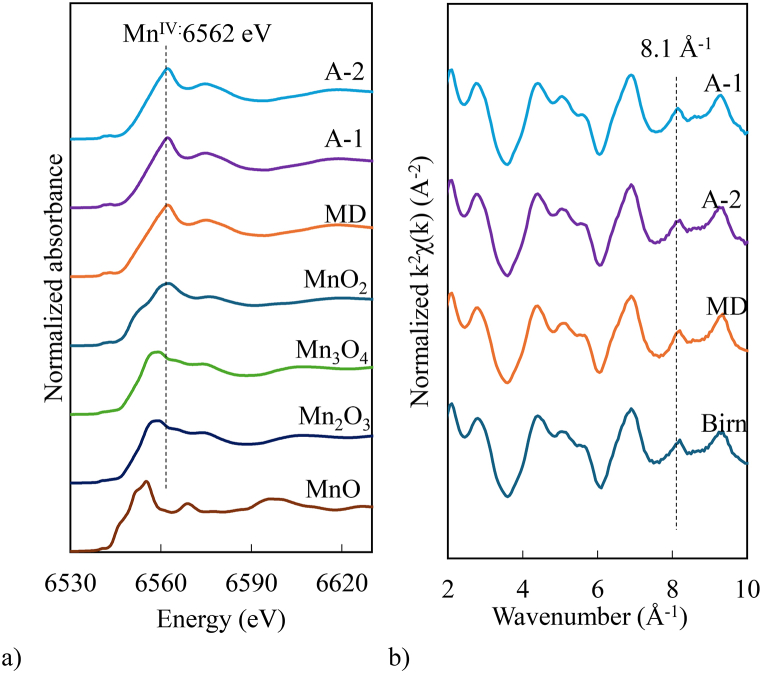
a) Mn K-edge XANES of the reference material and solid samples. Birn: birnessite; Mn K-edge XANES energy spectra for Mn oxidation state; Mn^II^ = 6555 eV, Mn^III^ = 6559 eV, Mn^IV^ = 6562 eV. b) Mn K-edge EXAFS spectra as normalised and background-subtracted k^2^ – weighted χ (k) (A^−2^) values of the reference material and solid samples.

The position of the white line peak in the Mn K-edge XANES spectra was used to determine the oxidation state of the Mn species in the solid samples (Fig. 4a). The photon energies of the XANES white-line peaks for MD, A-1, and A-2 were consistent at 6562 eV, which matched the MnO_2_ standard materials with an oxidation state of Mn^IV^. The Mn K-edge EXAFS spectra and Fourier transforms of the MD, A-1, and A-2 were highly similar, and their overall structures were similar to those of birnessite. These oscillations closely match the standard birnessite pattern, with a single antinode at k = ∼8.1 Å⁻^1^ (Fig. 4b), indicating that both birnessite in the MD and the pilot-scale passive treatment were the hexagonal birnessite type [37,38]. In the Fourier transforms of the EXAFS spectra, two prominent peaks were observed at R+△R 1.5 Å and 1.95 Å (Supplementary S3), which represented short elongated Mn-O and single scattering Mn1-O pairs within the octahedral unit and edge-sharing Mn - Mn pairs [38]. The radial distances R+△R 2.8 Å and 3.2 Å are recognised as corner-sharing Mn - Mn and Mn2-O shells [39]. The LCF of the Mn K-edge XANES (Supplementary S3) revealed that birnessite was the dominant form in the solid samples, accounting for 80.9 % in the MD, 96.5 % in A-1, and 93.7 % in A-2. Minor concentrations of Mn oxide minerals, including Mn_3_O_4_ and Mn_2_O_3_, suggested that Mn in the solid samples (mainly MD) existed in multiple oxidation states.

#### 3.3.2 XANES and EXAFS analysis of Zn compounds

**Fig. 5:**
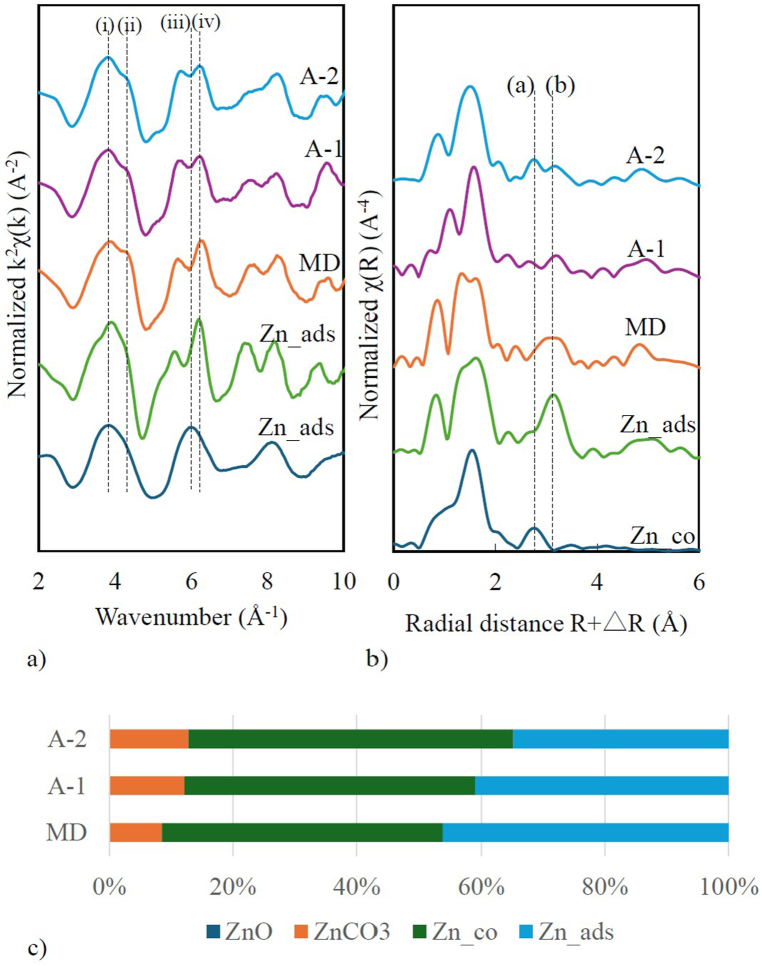
a) Zn K-edge EXAFS spectra as normalised and background-subtracted k^2^ – weighted χ (k) (A^−2^) values of the reference material and solid samples; (i) k = ∼3.8 Å^−1^, (ii) = ∼4.3 Å^−1^, (iii) k = ∼6.0 Å^−1^ , and (iv) k = ∼6.2 Å^−1^; b) Radal distribution function obtained from Fourier transforms of the Zn K-edge EXAFS spectra of the reference material and solid samples; (a) R+△R (Å) = ∼2.7 Å and (b) R+△R (Å) = ∼3.1 Å. c) Selective extraction from the solid sample and linear fitting combination (LCF) quantifying the mineral compositions in the solid samples using Zn- K-edge of XANES spectra energy.

The white line peak of the Zn K-edge XANES spectra for the MD, A-1, and A-2 was consistent at 9667 eV, which matched the Zn_Co, Zn_ads, and ZnCO_3_ standard materials with an oxidation state of Zn^II^ (Supplementary S3). The Zn K-edge EXAFS spectra and their Fourier transforms are presented in Fig. 5 and compared to the standard compound spectra. Woodruffite resulted from coprecipitation (Zn_co) mineral synthesis and shared the same chemical formula as ZnMn_3_O_7_ [40], which is similar to chacophanite (ZnMn_3_O_7_.3H_2_O), with an octahedral and tetrahedral Zn structure (^VI^Zn and ^IV^Zn on birnessite) [16,41]. The Zn_co reference sample's antinode at (i) k = ∼3.8 Å^−1^ and (iii) ∼6.0 Å^−1^ indicate ^VI^Zn, another antinode (ii) at k = ∼4.3 Å^−1^ and (iv) k = ∼6.2 Å^−1^ represent ^IV^Zn (Fig. 5a). These results suggest that the solid samples collected from the study area contained both ^VI^Zn and ^IV^Zn structure due to that adsorption and coprecipitation processes may be involved in Zn sequestration. In Fourier transforms of the EXAFS spectra, a prominent peak at R+△R 1.5 Å indicates the Zn-O shell pair in octahedral coordination (Fig. 5b), and it is located at the same position as the Mn-O pair in the octahedral unit. This indicates that Zn coordinated with one side to three O atoms of the Mn vacancy [41]. The coprecipitation Zn standard showed a peak at 2.7 Å (a), where Zn atoms directly associated with structural oxygen [42], and this value corresponds to the Zn-Mn interaction as tridentate edge-sharing. Another peak at R+△R 3.1 Å (b) represented a Zn-Mn adsorbed double corner (DC)/triple corner (TC) sharing complex above Mn vacancies in hexagonal birnessite [15,41]. The LCF of the Zn K-edge XANES spectra (Fig. 5c) revealed that the MD contains 45 % Zn adsorption and 46 % Zn coprecipitation (Fig. 5c). Zn coprecipitation increased slightly in the pilot treatment to approximately 47 % in A-1 and 52 % in A-2. The ZnCO_3_ concentrations were 8.5 %, 12.2 %, and 12.8 % in the MD, A-1, and A-2, respectively.

### 3.4 Mn^2+^ and Zn^2+^ removal mechanisms from the pilot-scale passive treatment

#### 3.4.1 Mn^2+^ treatment mechanisms

The chemical compositions obtained from XRF, and sequential extraction are listed in Supplementary S4. The total weight content of Mn in the MD, A-1, and A-2 were 45 %, 37 %, and 48 %, respectively. Sequential extraction of each fraction showed that up to 98 % was extracted from F2, which indicated Mn species in the natural sludge and treatment plant from the oxide phases (Supplementary S4). Birnessite was the main manganese oxide formed during the pilot-scale passive treatment process. In addition, the other possible minerals that formed were calculated based on the geochemical model. The results (attached in Supplementary S5) show that other MnO_2_ minerals formed, such as pyrolusite (S.I. = 8.96) and nutstite (S.I. = 7.78), which are found during Mn^2+^ removal from MD. However, birnessite is more common under biotic conditions at neutral pH conditions [43,44]. When birnessite is fully coated on limestone, the treatment process might be comparable to the implementation of Mn^2+^ treatment by the pyrolusite process [45,46]. However, in this system, bacteria seem to play a major role in Mn^2+^ oxidation [20]. This rapid oxidation of Mn^2+^ to Mn^4+^ is only possible when MnOB are catalysed under natural conditions [13,47]. Thus, Mn^2+^ was rapidly oxidised to Mn^4+^ in A-1 at circumneutral pH under biotic conditions (Reaction 1). MnOB in the limestone gravels in A-1 may have accelerated the Mn oxidation process, although no extra organic substrate was added to the treatment tanks. Heterotrophic Mn-oxidising communities may have yielded organic carbon from inorganic carbon when treatment tank A-1 was under organic substrate-limited conditions [17,20]. In addition, up to 90 % of MnO_2_ was found based on the XANES of the Mn K-edge spectra, whereas the other minerals were Mn_3_O_4_ and Mn_2_O_3_. Although MnCO_3_ is generated by during Mn^2+^ removal from the Mn^2+^ treatment of Mn-rich MD in limestone leach beds [48], this mineral was not found in the A-1 sludge. The autocatalytic process is also one of the Mn^2+^ treatment mechanisms owing to the adsorption of Mn^2+^, and the Mn oxide surface includes MnOOH, Mn_3_O_4_, MnO_2_, or MnO_x_ (Reaction 2); however, this process mainly occurs under alkaline conditions (pH < 7.8) [49,50]. In this study, the autocatalytic process did not appear to have any impact on the dissolved Mn^2+^ treatment mechanisms because of the low concentrations of Mn^2+^ species in the sludge. The passive treatment plant was operated under circumneutral conditions, where the Mn^2+^ oxidation process was catalysed by bacteria, which controlled the Mn removal mechanisms.

(1) Mn^2+^ + 1/2 O_2aq_ + H_2_O → MnO_2_ + 2H^+^

(2) Mn^2+^ + MnO_x_ + 1/2 O_2aq_ → 2MnO_x_

#### 3.4.2 Zn treatment mechanisms

The total concentration of Zn in the MD (6 %) was slightly lower than that in A-1 (9 %) and A-2 (13 %), whereas the total concentrations of Zn and Fe in the MD (7 %) were much higher than those in A-1 (1 %) and A-2 (0.23 %). Zn was found to be sequestered to the Mn oxide phase at 82 %, 94 %, and 75 % in the MD, A-1, and A-2, respectively, as it was extracted from F2 (Supplementary S4). A significant portion of Zn from sample A-2 was bound to F1 (approximately 25 %) owing to ion exchange or dissolution of ZnCO_3_. ZnCO_3_ was not detected by XRD but was detected in the Zn K-edge XANES spectra. In addition, ZnCO_3_ might have been oversaturated in the pilot system according to the S.I. (0.28) calculation. The limestone in treatment A-1 may have enhanced the availability of ZnCO_3_ in the passive treatment because it was detected in samples from A-1 and A-2 at a value of approximately 12 %. Zn did not show any significant incorporation into the Fe oxyhydroxide twisted stalk organo-mineral because the Zn concentration was very low in F3. Birnessite is a manganese oxide mineral with a high capacity for cation-surface complexation because of its large specific surface area [42,51]. Zn^2+^ has been reported to effectively sorb on the birnessite surface by up to 99 % at a pH greater than 4.5 [40,51,52]. Zn^2+^ was incorporated into birnessite as woodruffite in the pilot-scale passive treatment. Woodruffite has a tunnel structure similar to that of todorokite [53], which is formed by Zn^2+^ co-precipitation mechanisms with MnO_2_ at circumneutral pH [16,40]. Zn^2+^ also shows an affinity for binding with Mn^4+^ vacancy sites within hexagonal birnessite, creating inner-sphere surface complexes through tridentate edge sharing, DC, and TC sharing (Section 3.3.2). The LCF method determined that the co-precipitation mechanism was responsible for the removal of approximately 46.8 % while adsorption was responsible for approximately 41 %. Sequential extraction of the A-1 sludge showed that approximately 5 % of Zn^2+^ was in the exchangeable or soluble fraction while 94.2 % was in the reducible phase, which was incorporated into the birnessite structure. Moreover, Zn^2+^ and Mn^2+^ competition in the exchangeable phase (F1) might not have been influenced by the presence of Mn^2+^ in the pilot-scale passive treatment system because the Mn^2+^ content in F1 was only 0.03 %. At pH values greater than 6.5, competition between Zn^2+^ and Mn^2+^ generally does not occur [54]. In addition, although the rapid oxidation of Mn^2+^ to Mn^4+^ might prevent Mn^2+^ and Zn^2+^ competition, it enhanced Zn^2+^ structural incorporation into birnessite in the form of woodruffite. The MnOB community also had a secondary effect on woodruffite formation. The main mechanism responsible for Zn^2+^ removal in the pilot-scale passive treatment was incorporation into birnessite via two mechanisms: co-precipitation and adsorption at a ratio of approximately 1.25/1.

### 3.5 Numerical modelling of the pilot-scale passive treatment

The primary treatment mechanism occurred in the limestone tank in A-1, whereas the fibre filter acted as a filter medium to reduce the sludge particles and MnOB from discharging from A-2 at the treatment outlet. The Mn^2+^ and Zn^2+^ concentrations in A-2 were measured after the water was treated in A-1. Thus, an inverse model was constructed based on the measured data and treatment mechanisms in A-1.

#### 3.5.1 Inverse Mn^2+^ oxidation and transport

The water sample at MD created suitable conditions for the natural formation of bio-birnessite. Despite this natural attenuation capacity, the water quality in the MD remained higher than Japan's national effluent limit. However, limestone tank A-1 facilitated the Mn oxidation rate by promoting the growth of the Mn-oxidising bacteria [55]. Birnessite formation in the MD, A-1, and A-2 exhibited identical EXAFS and XANES patterns. This indicates that the formation of birnessite in A-1 and A-2 was the same as that in MD. However, a significant difference was observed between the oxidation rates of MD and A-1. The formation of birnessite in the MD was relatively slower than that in A-1 (Section 3.2). Compared with that in Mn oxidation under abiotic conditions, pH did not play a major role in controlling the oxidation rate in this pilot-scale passive treatment [11]. The bacterial species here highly control the oxidation rate because the limestone in tank A-1 provides a suitable environment for bacteria with sufficient dissolved oxygen provided by an air agitator [20]. The bacterial cell concentration in this study was unknown; thus, numerical modelling was used to simplify the characteristics of MnOB as a catalyser of Mn^2+^ oxidation (Reaction 1). Another mechanism involves Mn^2+^ adsorption on the MnO_2_ surface owing to an autocatalytic effect (Reaction 2) [56]. The overall Mn oxidation rate equation was provided by Morgan [49] (Equation (1)).

Equation 1 $$R_{Mn}=\frac{-d\left[{Mn}^{2+}\right]}{dt}=k_{1}\left[{Mn}^{2+}\right]+k_{2}\left[MnO_{x}\right]\left[{Mn}^{2+}\right]$$

Equation 2 $$R_{Mn}=\frac{-d\left[{Mn}^{2+}\right]}{dt}=k_{1}\left[{Mn}^{2+}\right]$$

where k_1_ is the pseudo-first-order reaction rate constant by dissolved Mn^2+^ disappearance from the solution and k_2_ is the autocatalytic reaction rate constant due at the mineral surface. Autocatalysis at the mineral surfaces and pH had less of an impact on Mn removal; therefore, the autocatalytic effect can be neglected [57]. The Mn^2+^ oxidation rate can be expressed as shown in Equation (2), which represents a homogeneous Mn^2+^ oxidation condition [49]. The oxidation rate constant k_1_ is important for understanding the Mn^2+^ oxidation rate in the pilot-scale treatment. Under abiotic conditions, k_1_ is relatively low and known as a slow reaction rate constant, whereas under biotic conditions, k_1_ is the rate constant of the bacterial cell concentration during the Mn^2+^ oxidation rate process [58,59]. The oxidation rate constant k_1_ is not universally uniform in the presence of MnOB. For instance, the Mn oxidation rate determined by Fuchida et al. [18] under biotic conditions using the rate law by Diem and Stumm [60] led to a misinterpretation of the pilot-scale passive treatment relative to the measured data (A-1). The oxidation rate constant in the presence of MnOB is highly uncertain because it is dependent on the Mn^2+^ concentration, pH, site-specific parameters, and bacterial cell concentrations [55,58]. Therefore, it is necessary to determine the value of the Mn oxidation rate constant of k_1_.

**Fig. 6:**
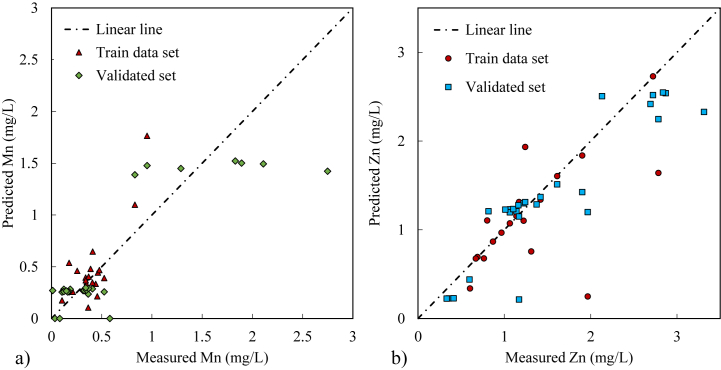
Comparison plots of measured data (x-axis) from the pilot-scale passive treatment against predicted data from the 1D reaction transport kinetics model (y-axis): a) Mn concentration, b) Zn concentration. The diagonal Linear line represents perfect prediction between measured and predicted data; R^2^ = 1.

The inverse model of the kinetic rate of Mn oxidation (Equation (2)) was constructed to obtained k_1_ as described in section 2.5. The result to the model calibration data was shown in Fig. 6a and Supplementary S6. The calibrated (trained data set) and validated data tend to align along the linear line when the Mn^2+^ concentration at A-1 is less than 0.5 mg/L with an HRT of 0.5 days. However, when the Mn^2+^ concentration at A-1 exceeds 0.5 mg/L (HRT of 0.3 days), the calibrated and validated data exhibit significant residuals (Fig. 6a). These large residuals may result from fluctuations in the natural mine drainage sources and the precipitation of minor Mn minerals other than birnessite (see Section 3.3). The selection of the rate constant, k_1_, was based on achieving the lowest NRMSE of 26 % among the evaluated k_1_ values of 1.03 × 10^−4^ s^−1^, which is approximately two to four order of magnitude greater than that of Fuchida et al. [18] (4.9 × 10^−7^ s^−1^) and Morgan (2005) (2.33 × 10^−8^ to 3.33 × 10^−6^ s^−1^) and approximately one of order magnitude greater than that of Zhang et al. [59] (2.78 × 10^−5^ s^−1^), as shown in Table 2. The experimental studies by Morgan [49] were conducted under abiotic conditions, which provided a smaller rate constant than that in this study, as expected. Fuchida et al. [18] and Zhang et al. [59] conducted studies under biotic conditions and calculated the Mn^2+^ oxidation rate constants using different equations. Thus, determining the factors that lead to Mn^2+^ oxidation rate constant variations is difficult. Zhang et al. [59] conducted an experiment in a controlled laboratory environment utilising the *Leptothrix discophora* SS1 bacterial species. In contrast, this study conducted an experiment in a natural setting at an average temperature of 15 °C and higher Mn^2+^ concentrations. The bacterial species in the pilot-scale passive treatment were likely a mixed culture of MnOB species, and the pH was slightly lower than that reported by Zhang et al. [59]. These different conditions may have led to variations in the Mn^2+^ oxidation rate constant observed in this and previous studies. The k_1_ value obtained in this study was higher than those obtained in previous studies. Factors such as the initial Mn^2+^ concentration, bacterial cell concentration, pH, and temperature at the treatment site may have increased the k_1_. Thus, it is crucial to consider these factors when determining the Mn^2+^ oxidation rate constant in an Mn^2+^ kinetic reaction model.

**Table 2:** Summary of the Mn^2+^ oxidation rate constants under different experimental conditions.

References	Water treatment	pH	Mn concentration	Calculated condition	Formula	Mn^2+^ oxidation rate constant (s^−1^)
This study	Passive treatment of mine wastewater	6.5–7.5	20 mg/L	Fitting methods of Mn^2+^ concentration in the pilot-scale treatment experiment using the rate law of Morgan [49].	$\frac{-d\left[{Mn}^{2+}\right]}{dt}=k_{1}\left[{Mn}^{2+}\right]$	1.03 × 10^−4^
Fuchida et al. [18]	Passive treatment of mine wastewater	6.0–8.0	0.2 mg/L	Fitting methods of Mn^2+^ concentration in the wetland's passive treatment using the rate law of Diem and Stumm [60]	$\frac{-d\left[{Mn}^{2+}\right]}{dt}=\left(k_{1}+k_{2}{\left[{\text{OH}}^{-}\right]}^{2}P_{O2}\right)\left[{\text{Mn}}^{2+}\right]$ PO2: oxygen partial pressure	4.90 × 10^−7^
Morgan [49]		8.0–9.3	2.7 mg/L	Experimental studies using pseudo first-order homogeneous solution	$\frac{-d\left[{Mn}^{2+}\right]}{dt}=k_{1}\left[{Mn}^{2+}\right]$	2.33 × 10^−8^ to 3.33 × 10^−6^
Zhang et al. [59]		7.5–8.0	2.7 mg/L	Bacteria oxidation *Leptothrix discophora* SS1 using natural water conditions of the Black Sea	$\frac{-d\left[{Mn}^{2+}\right]}{dt}=\frac{k\left[X\right]\left[{Mn}^{2+}\right]}{K_{s}+\left[{Mn}^{2+}\right]}$ k: Mn^2+^ oxidation rate constant; [X]: cell concetration (mg/L); K_s_: half velocity constant (mol Mn^2+^/mg cell. s)	1.11 × 10^−5^ to 2.78 × 10^−5^
Davies and Morgan [57]		8.0	2.7 mg/L	Experimental study under metals oxide surfaces catalysis	$\frac{-d\left[{Mn}^{2+}\right]}{dt}=\frac{k^{\prime \prime }\left\{>SOH\right\}\;\left[{Mn}^{2+}\right]}{{\left[H^{+}\right]}^{2}}AP_{O2}$ k”: surface rate constant (s^−1^ atm^−1^); {>SOH} surface concentration (mol/g), A: solid concentration (g/L)	2.77 × 10^−7^

### 3.6 Inverse sorption kinetics of Zn^2+^ and transport

Zn^2+^ incorporated with birnessite via adsorption and coprecipitation mechanisms at a similar portion; thus, to simplify the mechanisms, the Zn^2+^ sorption kinetic reaction (R_Zn_) on birnessite was determined using the approach outlined by Tebes-Stevens and Valocchi [61,62], as shown in Equation (3).

Equation (3) $$R_{Zn}=-k_{m}\left(c_{i}-\frac{s_{Zn}}{K_{d}}\right)$$

where S_Zn_ represents the mass of the sorbed concentration (mol/g of sediment), k_m_ represents the mass-transfer coefficient (s^−1^), and K_d_ represents the distribution coefficient (L/g). k_m_ and K_d_ are typically determined by column or batch experiments; however, in this study, an experiment to obtain k_m_ and K_d_ was not implemented. Studies on the k_m_ of Zn immobilisation and sorption on birnessite are limited. Thus, the use of an inverse model to determine k_m_ is suggested if experiments are not performed [62,63].

An inverse model of the Zn^2+^ sorption reaction 1D transport by PHREEQC was constructed to obtain the k_m_ and K_d_ values of Zn^2+^ sorption on birnessite (The detailed model description was attached in Supplementary S6, and section 2.5). Fig. 6b presents both the calibrated and validated datasets. The calibrated data align closely with the linear trend, except during periods of increased Zn^2+^ concentrations at the input source (A-0). Similarly, the validated dataset exhibits smaller residuals compared to the Mn concentration results (Section 3.5.1). For the kinetic and transport models within the passive treatment with lowest NRMSE of k_m_ and K_d_ was 20 % and the values of k_m_ and K_d_ were 4.11 × 10^4^ s^−1^ (11.43 h^−1^) and 4.49 L/g, respectively. k_m_ is controlled by the physicochemical conditions of the transport medium [64]. Tebes-Stevens et al. [65] reported that k_m_ ranges from 1 h^−1^ to 100 h^−1^ to reach the equilibrium sorption reactions. The smaller time step may provide a larger k_m_ value even though k_m_ might be affected by the dispersion and diffusion of the numerical model dimensions [62,65,66]. In addition, K_d_ can be calculated using the Freundlich or Langmuir curve fit, although it is dependent on the initial concentration of Zn [67,68]. For example, the Langmuir constant (K) of 54.93 mg/L Zn (1 mM) absorption on birnessite (1/500 w/v ratio) based on the kinetic batch experiment was 0.38 L/g at pH 5.5 [14]. K_d_ value was in the range found in literature review, but the pH in this study was relatively higher (7.5), which may effect on the Zn sorption capacity and K_d_ [41,69].

#### 3.6.1 HRT optimisation for passive treatment

The HRT in the pilot-scale experiment was changed under various conditions, although the optimum HRT has not yet been determined. The obtained Mn^2+^ and Zn^2+^ reaction transport kinetics parameters (k_1_, k_m_, and K_d_) from inverse modelling were reconstructed to predict the Mn^2+^ and Zn^2+^ concentrations in the pilot-scale treatment. The 1D reaction transport model of Mn^2^^+^ and Zn^2^^+^ is used to determine the optimum HRT for the pilot-scale passive treatment based on the obtained parameters; k_1_ = 1.03 × 10^−4^ s^−1^, k_m_ = 4.11 × 10^4^ s^−1^, and K_d_ = 4.49 L/g. Firstly, the predicted model of Mn^2^^+^ and Zn^2^^+^ concentrations is used to compare with the field monitoring data from 2022. Later, the predictive model is used to obtain the optimum HRT for pilot-scale treatment.

**Fig. 7:**
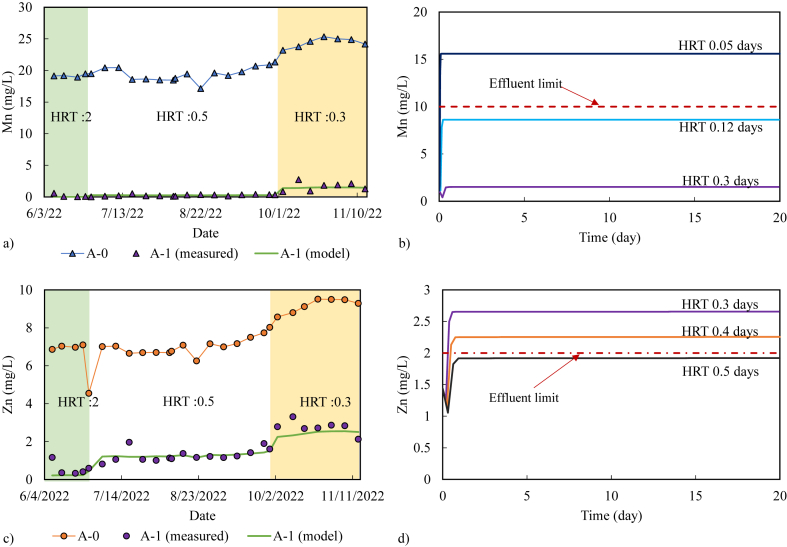
Reaction transport kinetics of Mn^2+^ and Zn^2+^ in the pilot-scale passive treatment. a) Mn concentration calculated from the model and Mn^2+^ concentration from field measurements of A-1; b) predictive model of the Mn^2+^ concentration in relation to the HRT (day) using the maximum concentration of Mn^2+^ of 25 mg/L; c) Zn^2+^ concentration calculated from the model and Zn^2+^ concentration from field measurement of A-1; d) predictive model of the Zn^2+^ concentration in relation to the HRT using the maximum concentration of Zn^2+^ 9.5 mg/L.

The obtained k_1_ was used in the forward 1D reaction transport model to predict Mn^2+^ concentration from model simulations. The comparison between the Mn^2+^ predicted by the model and the Mn^2+^ monitoring data of A-1 in 2022 is shown in Fig. 7a. The overall MAE and RMSE of Mn^2+^ in the predictive model and monitoring data were 0.24 mg/L and 0.37 mg/L, respectively, indicating that the predicted model error is within ±13.4 % (Supplementary S7). The predicted data with various HRTs (2 days, 0.5 days, and 0.3 days) were investigated to observe the model prediction accuracy at each HRT. The HRT of 0.3 days provided the largest error of ±23.9 %. This is probably due to the increase in Mn concentration from 20 mg/L to 25 mg/L at the inlet of the treatment plan (A-0), while the calibration dataset used the Mn^2+^ input from A-0 in 2021 with an Mn^2+^ concentration of about 15 mg/L [20]. Additionally, there were abnormal Mn concentrations in the monitoring data (HRT 0.3 days in 2022) with Mn values of 2.75 mg/L and 0.95 mg/L, while other Mn concentrations ranged from 1.3 to 2.1 mg/L (Fig. 7a). It is possible that the fluctuation in Mn concentration may affect the predictive model error. Even so, this predictive model is reliable up to 74 % (Mn_R^2^ = 0.74).

Similarly, the k_m_ and K_d_ values were applied to the forward reaction transport model. The MAE and RMSE of the Zn^2^⁺ concentrations relative to the predictive model at an HRT of 0.5 days had the lowest values of 0.19 mg/L and 0.27 mg/L, respectively, while larger variations were observed at HRTs of 2 and 0.3 days (Fig. 7c). The MAE and RMSE of the overall model prediction were 0.27 mg/L and 0.38 mg/L, respectively, with an overall NRMSE of ±12.6 % and a Zn_R^2^ of 0.80 (Supplementary S7). A larger error occurred owing to the instability of the Zn^2+^ sorption mechanisms in the limestone tank and the Zn^2+^ concentration at the input of A-0. k_m_ and K_d_ under natural conditions are more complex, particularly under microscale conditions [28,70,71]. The sorption kinetics and 1D transport model in PHREEQC may not be able to represent the actual boundary conditions as well as the 3D groundwater flow and transport model, where diffusion, dispersion, and advection are better represented [25,72]. However, it is worth mentioning that the goodness-of-fit of the model was up to 80 %.

The goodness-of-fit (R^2^: 0.74–0.80) of the predictive model of Mn^2^^+^ and Zn^2^^+^ concentrations indicates that up to 74 % and 80 % of the predictive model of Mn^2^^+^ and Zn^2^^+^ can be explained by the monitoring data [27]. Additionally, the NRMSE of the predictive model clearly indicates a possible error of ±13 % between the predictive model and the monitoring data. In the pilot-scale passive treatment, HRT at 0.3 day corresponded to the largest flow rate in the onsite experiment. In the optimised HRT model, the flowrate of the reconstructed model was adjusted to meet an HRT 0.1 days and 0.05 days with a maximum Mn^2+^ concentration of 25 mg/L. Consequently, an HRT 0.05 days did not meet the Mn effluent limit (Fig. 7b). To treat Mn^2+^ at the concentration in the pilot treatment, it was important to maintain an HRT of more than 0.12 days.

Similarly, the reconstructed model realised Zn^2+^ treatment efficiency in the pilot-scale treatment with different HRTs (Fig. 7d). An HRT of 0.3 days was not able to achieve Zn^2+^ sorption onto birnessite and did not meet the effluent limit requirement. The Zn^2+^ sorption transport model suggests that successful Zn^2+^ treatment can be achieved only when the HRT in the pilot-scale passive treatment exceeds 0.4 days when the average inlet Zn^2+^ concentration at A-0 is less than 8.88 mg/L. However, the maximum Zn^2+^ concentration recorded during field measurements at A-0 was 9.5 mg/L. The Zn^2+^ treatment capacity of the pilot-scale treatment was less than that of the Mn^2+^ treatment because MnOB highly controlled Mn^2+^ in the passive treatment, whereas Zn^2+^ was particularly dependent on the formation of birnessite. The pilot-scale passive treatment was successful because it was mainly controlled by the biological conditions for Mn and geochemical conditions for Zn^2+^. Here, Zn^2+^ is the rate-limiting process; therefore, to ensure that both Mn^2+^ and Zn^2+^ are reduced below the effluent limit, an HRT of 0.5 days is recommended. An HRT of 0.5 days will allow for the successful passive treatment of both Mn^2+^ and Zn^2+^, even if there are seasonal fluctuations.

## 4 Conclusion

The pilot-scale passive treatment system was effectively operated within a pH range of 6.5–7.5 and using MD with Mn/Zn mole ratios of 2.5. A-1 and A-2 demonstrated significant Mn^2+^ removal efficiency, with removal rates of 98 % at HRTs of 2 and 0.5 days, indicating the successful removal of Mn^2+^ from the system. However, the removal rate decreased to 91 % at an HRT of 0.3 days, despite Mn^2+^ removal meeting the national effluent limit. The behaviour of Zn^2+^ removal was different, with Zn^2+^ removal rates of 94 %, 82 %, and 69 % for HRTs in A-1 of 2, 0.5, and 0.3 days, respectively. The decrease in Zn^2+^ removal efficiency with a shorter HRT highlights the importance of the HRT in the treatment process. Analysis of the sludge revealed the presence of birnessite, woodruffite, and Fe oxyhydroxide twisted stalks. However, only birnessite and woodruffite were responsible for the removal of Mn^2+^ and Zn^2+^ during the pilot treatment. Notably, Mn^2+^ and Zn^2+^ did not bind to the Fe oxyhydroxide twisted stalks.

Geochemical and inverse kinetic transport analyses can successfully determine critical parameters, including the Mn^2+^ oxidation rate constant (k_1_), mass transfer coefficient (k_m_), and distribution coefficient (K_d_), and they do not require additional experiments. This information was instrumental in the numerical modelling process, which allowed for the effective reconstruction of the pilot-scale experiment. This modelling not only provided insights into the system's behaviour but also optimised the suitable HRTs for Mn^2+^ and Zn^2+^ removal from mine drainage. Moreover, the HRT emerged as a critical factor, and an HRT of 0.5 days was particularly applicable for achieving optimal removal rates for both Mn^2+^ and Zn^2+^.

In summary, the effectiveness of the proposed pilot-scale passive treatment system was demonstrated, and the geochemical analysis and numerical modelling provided insights towards establishing a foundation for further refining and optimising the treatment process. Thus, the proposed method offers promising solutions for Mn^2+^ and Zn^2+^ removal from mine drainage. The values obtained for k_1_, k_m_, and K_d_ from the 1D inverse Mn^2+^ and Zn^2+^ kinetic reaction transport models for the pilot-scale treatment remained limited, and the RMSE of the 1D model may be reduced by further parameter optimisation using a 3D model that considers other boundary conditions. Utilising the inverse numerical model can result in significant time and cost savings compared with traditional laboratory experiments.

## CRediT authorship contribution statement

**Sereyroith Tum:** Writing – review & editing, Writing – original draft, Visualization, Validation, Methodology, Investigation, Formal analysis, Data curation. **Taiki Katayama:** Writing – review & editing, Validation, Investigation, Conceptualization. **Naoyuki Miyata:** Writing – review & editing, Validation, Methodology, Investigation, Formal analysis, Conceptualization. **Miho Watanabe:** Writing – review & editing, Investigation, Conceptualization. **Yohey Hashimoto:** Writing – review & editing, Validation, Formal analysis. **Miu Nishikata:** Writing – review & editing, Investigation, Formal analysis. **Tetsuo Yasutaka:** Writing – review & editing, Validation, Supervision, Project administration, Funding acquisition, Conceptualization.

## Data availability statement

The raw data required to reproduce the above findings are available in Supplementary S6.

## Funding sources

This study was funded by Ministry of Economy, Trade, and Industry, Japan (METI) under the research project on Advanced Technology for Mine Drainage Treatment in Closed Mines for the year 2023. The funders had no role in the study design, data collection, data analysis, or decision to publish.

## Declaration of competing interest

The authors declare that they have no known competing financial interests or personal relationships that could have appeared to influence the work reported in this paper.

## Footnotes

Appendix A Supplementary data to this article can be found online at https://doi.org/10.1016/j.heliyon.2024.e40363.

## References

[1] Chikanda F., Tum S., Matsui T., Norota S., Otake T., Sato T. (Jan. 2023). The formation of schwertmannite colloids and natural remediation of toxic elements from S hojin R iver, H okkaido, J apan. Resour. Geol..

[2] Outram J.G., Couperthwaite S.J., Millar G.J. (Apr. 2018). Enhanced removal of high Mn(II) and minor heavy metals from acid mine drainage using tunnelled manganese oxides. J. Environ. Chem. Eng..

[3] Tum S. (Feb. 2022). Seasonal effects of natural attenuation on drainage contamination from artisanal gold mining, Cambodia: implication for passive treatment. Sci. Total Environ..

[4] Obasi P.N., Akudinobi B.B. (Jul. 2020). Potential health risk and levels of heavy metals in water resources of lead–zinc mining communities of Abakaliki, southeast Nigeria. Appl. Water Sci..

[5] Turdiyeva K., Lee W. (Jun. 2023). Comparative analysis and human health risk assessment of contamination with heavy metals of Central Asian rivers. Heliyon.

[6] Ford K.L. (2003).

[7] Skousen J. (Mar. 2017). Review of passive systems for acid mine drainage treatment. Mine Water Environ..

[8] Patil D.S., Chavan S.M., Oubagaranadin J.U.K. (Mar. 2016). A review of technologies for manganese removal from wastewaters. J. Environ. Chem. Eng..

[9] Romano C.A. (Dec. 2017). Biogenic manganese oxide nanoparticle formation by a multimeric multicopper oxidase Mnx. Nat. Commun..

[10] Feng X.H., Zhai L.M., Tan W.F., Liu F., He J.Z. (May 2007). Adsorption and redox reactions of heavy metals on synthesized Mn oxide minerals. Environ. Pollut..

[11] Li Y., Xu Z., Ma H., Hursthouse A.S. (Nov. 2019). Removal of manganese(II) from acid mine wastewater: a review of the challenges and opportunities with special emphasis on Mn-oxidizing bacteria and microalgae. Water.

[12] Barboza N.R., Morais M.M.C.A., Queiroz P.S., Amorim S.S., Guerra-Sá R., Leão V.A. (Oct. 2017). High manganese tolerance and biooxidation ability of Serratia marcescens isolated from manganese mine water in minas gerais, Brazil. Front. Microbiol..

[13] Dangeti S., McBeth J.M., Roshani B., Vyskocil J.M., Rindall B., Chang W. (Mar. 2020). Microbial communities and biogenic Mn-oxides in an on-site biofiltration system for cold Fe-(II)- and Mn(II)-rich groundwater treatment. Sci. Total Environ..

[14] Della Puppa L., Komárek M., Bordas F., Bollinger J.-C., Joussein E. (Jun. 2013). Adsorption of copper, cadmium, lead and zinc onto a synthetic manganese oxide. J. Colloid Interface Sci..

[15] Hinkle M.A.G., Dye K.G., Catalano J.G. (Mar. 2017). Impact of Mn(II)-Manganese oxide reactions on Ni and Zn speciation. Environ. Sci. Technol..

[16] Tajima S., Fuchida S., Tokoro C. (Nov. 2022). Coprecipitation mechanisms of Zn by birnessite formation and its mineralogy under neutral pH conditions. J. Environ. Sci..

[17] Miyata N. (Mar. 2024). Biological Mn(II) oxidation under organic substrate-limited conditions and its application in mine drainage remediation. Biochem. Eng. J..

[18] Fuchida S., Tajima S., Nishimura T., Tokoro C. (Jan. 2022). Kinetic modeling and mechanisms of manganese removal from alkaline mine water using a pilot scale column reactor. Minerals.

[19] Yamamoto G., Nagamine T., Kitagaki T., Unno T. (2004). Discovery of the manganese nodule in ground water from the oppu mine, aomori prefecture, Japan. Earth Sci. Chikyu Kagaku.

[20] Watanabe M. (Oct. 2024). Accelerated manganese(II) removal by in situ mine drainage treatment system without organic substrate amendment: metagenomic insights into chemolithoautotrophic manganese oxidation via extracellular electron transfer. J. Environ. Chem. Eng..

[21] Yamamoto K., Hashimoto Y., Kang J., Kobayashi K. (Nov. 2018). Speciation of phosphorus zinc and copper in soil and water-dispersible colloid affected by a long-term application of swine manure compost. Environ. Sci. Technol..

[22] Pueyo M., Mateu J., Rigol A., Vidal M., López-Sánchez J.F., Rauret G. (Mar. 2008). Use of the modified BCR three-step sequential extraction procedure for the study of trace element dynamics in contaminated soils. Environ. Pollut..

[23] Postma D., Appelo C.A.J. (Apr. 2000). Reduction of Mn-oxides by ferrous iron in a flow system: column experiment and reactive transport modeling. Geochem. Cosmochim. Acta.

[24] Swiler L., Wyss G. (Jul. 2004).

[25] Knowling M.J., Werner A.D. (Sep. 2016). Estimability of recharge through groundwater model calibration: insights from a field-scale steady-state example. J. Hydrol..

[26] Klaas N., Faber M. (Sep. 1999). Estimating the uncertainty in estimates of root mean square error of prediction: application to determining the size of an adequate test set in multivariate calibration. Chemometr. Intell. Lab. Syst..

[27] Legates D.R., McCabe G.J. (Jan. 1999). Evaluating the use of ‘goodness‐of‐fit’ Measures in hydrologic and hydroclimatic model validation. Water Resour. Res..

[28] Nakamura K., Yasutaka T., Kuwatani T., Komai T. (Nov. 2017). Development of a predictive model for lead, cadmium and fluorine soil–water partition coefficients using sparse multiple linear regression analysis. Chemosphere.

[29] Picard A., Kappler A., Schmid G., Quaroni L., Obst M. (Feb. 2015). Experimental diagenesis of organo-mineral structures formed by microaerophilic Fe(II)-oxidizing bacteria. Nat. Commun..

[30] Suzuki T., Hashimoto H., Matsumoto N., Furutani M., Kunoh H., Takada J. (May 2011). Nanometer-scale visualization and structural analysis of the inorganic/organic hybrid structure of gallionella ferruginea twisted stalks. Appl. Environ. Microbiol..

[31] Li Y., Jiang G., Ouyang N., Qin Z., Lan S., Zhang Q. (May 2021). The controlled synthesis of birnessite nanoflowers via H2O2 reducing KMnO4 for efficient adsorption and photooxidation activity. Front. Chem..

[32] Zhu H.T. (Nov. 2008). Birnessite-type MnO _2_ nanowalls and their magnetic properties. J. Phys. Chem. C.

[33] Kumpulainen S., Carlson L., Räisänen M.-L. (Apr. 2007). Seasonal variations of ochreous precipitates in mine effluents in Finland. Appl. Geochem..

[34] Lu J., Walder I., Leiviskä T. (Dec. 2021). Impact of temperature on the leaching of sulphate, Co, Fe, Mn, Ni and Zn from the Ballangen tailings deposit, Norway: a laboratory column experiment. J. Water Clim. Change.

[35] Tum S., Matsumoto S., Nishikata M., Yasutaka T. (Mar. 2023). Assessment of seasonal changes in groundwater quality of waste rock dump in temperate continental climate, northern Japan. Chemosphere.

[36] National Effluent Standards,” Ministry of the Environment, Government of Japan. Accessed: August. 16, 2022. [Online]. Available: https://www.env.go.jp/en/water/wq/nes.html.

[37] Ling F.T., Post J.E., Heaney P.J., Ilton E.S. (Feb. 2018). The relationship between Mn oxidation state and structure in triclinic and hexagonal birnessites. Chem. Geol..

[38] Yin H. (Mar. 2018). Coordination geometry of Zn2+ on hexagonal turbostratic birnessites with different Mn average oxidation states and its stability under acid dissolution. J. Environ. Sci..

[39] Qin Z. (Feb. 2019). Effect of γ-manganite particle size on Zn2+ coordination environment during adsorption and desorption. Appl. Clay Sci..

[40] Chang J., Tani Y., Naitou H., Miyata N., Tojo F., Seyama H. (Sep. 2014). Zn(II) sequestration by fungal biogenic manganese oxide through enzymatic and abiotic processes. Chem. Geol..

[41] Wang Z., Peacock C., Kwon K.D., Gu X., Feng X., Li W. (May 2023). Site-specific isotope fractionation during Zn adsorption onto birnessite: insights from X-ray absorption spectroscopy, density functional theory and surface complexation modeling. Geochem. Cosmochim. Acta.

[42] Appelo C.A.J., Postma D. (Oct. 1999). A consistent model for surface complexation on birnessite (−MnO2) and its application to a column experiment. Geochem. Cosmochim. Acta.

[43] Tan H., Zhang G., Heaney P.J., Webb S.M., Burgos W.D. (Mar. 2010). Characterization of manganese oxide precipitates from Appalachian coal mine drainage treatment systems. Appl. Geochem..

[44] Tebo B.M. (May 2004). Biogenic manganese oxides: properties and mechanisms of formation. Annu. Rev. Earth Planet Sci..

[45] Christenson H. (Apr. 2019). Manganese and trace element removal from New Zealand coal mine drainage using limestone leaching beds. N. Z. J. Geol. Geophys..

[46] Neculita C.M., Rosa E. (Jan. 2019). A review of the implications and challenges of manganese removal from mine drainage. Chemosphere.

[47] Bohu T., Akob D.M., Abratis M., Lazar C.S., Küsel K. (May 2016). Biological low-pH Mn(II) oxidation in a manganese deposit influenced by metal-rich groundwater. Appl. Environ. Microbiol..

[48] Okibe N., Nonaka K., Kondo T., Shimada K., Liu P. (May 2023). Microbiological passive treatment of Mn/Zn-containing mine water. Hydrometallurgy.

[49] Morgan J.J. (Jan. 2005). Kinetics of reaction between O2 and Mn(II) species in aqueous solutions. Geochem. Cosmochim. Acta.

[50] Morgan J.J., Schlautman M.A., Bilinski H. (Nov. 2021). Rates of abiotic Mn ^II^ oxidation by O _2_ : influence of various multidentate ligands at high pH. Environ. Sci. Technol..

[51] Tonkin J.W., Balistrieri L.S., Murray J.W. (Jan. 2004). Modeling sorption of divalent metal cations on hydrous manganese oxide using the diffuse double layer model. Appl. Geochem..

[52] Toner B., Manceau A., Webb S.M., Sposito G. (Jan. 2006). Zinc sorption to biogenic hexagonal-birnessite particles within a hydrated bacterial biofilm. Geochem. Cosmochim. Acta.

[53] Post J.E., Heaney P.J., Cahill C.L., Finger L.W. (Nov. 2003). Woodruffite: a new Mn oxide structure with 3 × 4 tunnels. Am. Mineral..

[54] Lefkowitz J.P., Elzinga E.J. (Apr. 2015). Impacts of aqueous Mn(II) on the sorption of Zn(II) by hexagonal birnessite. Environ. Sci. Technol..

[55] Bruins J.H. (Feb. 2015). Biological and physico-chemical formation of Birnessite during the ripening of manganese removal filters. Water Res..

[56] Learman D.R., Wankel S.D., Webb S.M., Martinez N., Madden A.S., Hansel C.M. (Oct. 2011). Coupled biotic–abiotic Mn(II) oxidation pathway mediates the formation and structural evolution of biogenic Mn oxides. Geochem. Cosmochim. Acta.

[57] Davies S.H.R., Morgan J.J. (Apr. 1989). Manganese(II) oxidation kinetics on metal oxide surfaces. J. Colloid Interface Sci..

[58] Godwin C.M., Zehnpfennig J.R., Learman D.R. (May 2020). Biotic and abiotic mechanisms of manganese (II) oxidation in lake erie. Front. Environ. Sci..

[59] Zhang J., Lion L.W., Nelson Y.M., Shuler M.L., Ghiorse W.C. (Mar. 2002). Kinetics of Mn(II) oxidation by Leptothrix discophora SS1. Geochem. Cosmochim. Acta.

[60] D. Diem and W. Stumm, “Is Dissolved Mn2+ Being Oxidized by O2 in Absence of Mn-Bacteria or Surface Catalysts?”.

[61] Parkhurst D.L., Appelo C.A.J. (2013). Techniques and Methods.

[62] Tebes-Stevens C., Valocchi A.J., VanBriesen J.M., Rittmann B.E. (Aug. 1998). Multicomponent transport with coupled geochemical and microbiological reactions: model description and example simulations. J. Hydrol..

[63] Nuić I., Trgo M., Vukojević Medvidović N., Ugrina M. (Feb. 2019). A mass transfer analysis of competitive binding of Pb, Cd, and Zn from binary systems onto a fixed zeolite bed. Int. J. Environ. Res. Publ. Health.

[64] Greskowiak J. (Sep. 2010). Comparison of parameter sensitivities between a laboratory and field‐scale model of uranium transport in a dual domain, distributed rate reactive system. Water Resour. Res..

[65] Tebes-Stevens C.L., Espinoza F., Valocchi A.J. (Nov. 2001). Evaluating the sensitivity of a subsurface multicomponent reactive transport model with respect to transport and reaction parameters. J. Contam. Hydrol..

[66] Pei J., Zhang J. (Mar. 2010). Modeling of sorbent-based gas filters: development, verification and experimental validation. Build. Simulat..

[67] Antoniadis V., McKinley J.D., Zuhairi W.Y.W. (Jan. 2007). Single‐Element and competitive metal mobility measured with column infiltration and batch tests. J. Environ. Qual..

[68] Behroozi A., Arora M., Fletcher T.D., Western A.W. (May 2020). Sorption and transport behavior of zinc in the soil; Implications for stormwater management. Geoderma.

[69] Li Y., Zhao X., Wu J., Gu X. (Sep. 2020). Surface complexation modeling of divalent metal cation adsorption on birnessite. Chem. Geol..

[70] Kent D.B., Abrams R.H., Davis J.A., Coston J.A., LeBlanc D.R. (Dec. 2000). Modeling the influence of variable pH on the transport of zinc in a contaminated aquifer using semiempirical surface complexation models. Water Resour. Res..

[71] Trgo M., Perić J., Medvidović N.V. (May 2006). Investigations of different kinetic models for zinc ions uptake by a natural zeolitic tuff. J. Environ. Manag..

[72] Dettrick D., Costelloe J., Arora M., Yuen S. (Feb. 2019). A comparison of measured and predicted diffusion coefficients applied to sand and silt sized acid mine drainage materials. J. Environ. Manag..

