# Supplementary material for: Geochemical insights and model optimisation for pilot-scale passive treatment of manganese and zinc in a legacy mine in Japan
Source: Heliyon. 2024 Nov 13;10(22):e40363. doi: 10.1016/j.heliyon.2024.e40363 (PMC11616510; doi:10.1016/j.heliyon.2024.e40363)
Supplement: Multimedia component 1 [file mmc1.docx]

Supplementary data of Geochemical insights and model optimisation for pilot-scale passive treatment of manganese and zinc in a legacy mine in Japan

Sereyroith Tum^a^, Taiki Katayama^a^, Naoyuki Miyata^b^, Miho Watanabe^b^, Yohey Hashimoto^c^, Miu Nishikata^a^, and Tetsuo Yasutaka^a*^

^a^Institute for Geo-Resources and Environment, Geological Survey of Japan, National Institute of Advanced Industrial Science and Technology (AIST), 1-1-1 Central 7, Higashi, 305-8567, Tsukuba, Ibaraki, Japan

^b^Department of Biological Environment, Akita Prefectural University, 241-438, Shimoshinjo-Nakano, 010-0195, Akita, Japan

^c^Department of Bioapplications and Systems Engineering (BASE), Tokyo University of Agriculture and Technology, 2-24-16, Nakamachi Koganei,184-8588, Tokyo, Japan

*Correspondence: Tetsuo Yasutaka; [t.yasutaka@aist.go.jp](mailto:t.yasutaka@aist.go.jp)

# Supplementary S1

Table S1. Description of sequential extraction fractionation using BCR method by Pueyo et al. [1].

| **Fraction** | **Solutions** | **Targets** |
| --- | --- | --- |
| F1 | Acetic acid (0.11 mol/L) | Extracts the water, acid-soluble or ion exchangeable metals |
| F2 | Hydroxylammonium chloride (0.5 mol/L) | Iron/manganese oxides minerals phase |
| F3 | Hydrogen peroxide (8.8 mol/L, 30%), D: Ammonium acetate (1 mol/L), adjusting the pH to 2 with HNO_3_. | Organic matter or the oxidisable minerals. |

Table S2. The input 1D transport parameters of A-1 and initial and boundary conditions

| **Property** | **Value** | **Unit** |
| --- | --- | --- |
| Length of pilot scale tank | 1.6 | m |
| Cell | 16 | - |
| Length | 0.1 | m |
| Porosity | 42 | % |
| Grams of sediment per liter | 3.75 ×10^3^ | g/L |
| Bulk density limestone | 2.7 | g/m^3^ |
| Longitudinal dispersity | 0.05 | m |

Table S3. The concetration of the Mn^2+^ and Zn^2+^ input Phreeqc and calibrated data for PEST computer code.

| **Mn (mg/L)** | **Mn (mg/L)** | **Zn (mg/L)** | **Zn (mg/L)** | **Year** | **HRT** |
| --- | --- | --- | --- | --- | --- |
| Inlet (A-0) | Calibrated (A-1) | Inlet | Calibrated (A-1) |  | day |
| 13.81 | 0.20 | 5.53 | 0.80 | 2021 | 1 |
| 14.23 | 0.35 | 5.49 | 1.06 | 2021 | 1 |
| 15.18 | 0.19 | 5.75 | 0.83 | 2021 | 1 |
| 15.02 | 0.21 | 5.59 | 0.78 | 2021 | 1 |
| 15.73 | 0.21 | 5.95 | 0.87 | 2021 | 0.5 |
| 15.43 | 0.46 | 5.65 | 0.75 | 2021 | 0.5 |
| 16.58 | 0.44 | 6.01 | 0.69 | 2021 | 0.5 |
| 15.18 | 0.46 | 5.80 | 0.67 | 2021 | 0.5 |
| 16.63 | 0.48 | 6.06 | 0.60 | 2021 | 0.5 |
| 14.84 | 0.39 | 5.88 | 0.97 | 2021 | 0.5 |
| 19.51 | 0.01 | 4.55 | 0.60 | 2022 | 0.51 |
| 20.44 | 0.12 | 7.02 | 0.82 | 2022 | 0.51 |
| 20.46 | 0.19 | 7.04 | 1.07 | 2022 | 0.51 |
| 18.60 | 0.53 | 6.65 | 1.97 | 2022 | 0.51 |
| 18.66 | 0.18 | 6.69 | 1.06 | 2022 | 0.51 |
| 18.50 | 0.16 | 6.70 | 1.01 | 2022 | 0.51 |
| 18.47 | 0.10 | 6.69 | 1.14 | 2022 | 0.51 |
| 18.76 | 0.12 | 6.77 | 1.10 | 2022 | 0.51 |
| 19.46 | 0.32 | 7.09 | 1.37 | 2022 | 0.51 |
| 17.16 | 0.37 | 6.25 | 1.17 | 2022 | 0.51 |
| 19.62 | 0.33 | 7.16 | 1.22 | 2022 | 0.51 |
| 19.22 | 0.15 | 7.00 | 1.16 | 2022 | 0.51 |
| 19.80 | 0.35 | 7.17 | 1.24 | 2022 | 0.51 |
| 20.71 | 0.41 | 7.50 | 1.42 | 2022 | 0.51 |
| 20.91 | 0.37 | 7.74 | 1.90 | 2022 | 0.51 |
| 21.35 | 0.34 | 8.03 | 1.61 | 2022 | 0.51 |
| 23.21 | 0.83 | 8.57 | 2.78 | 2022 | 0.31 |
| 23.76 | 2.75 | 8.81 | 3.31 | 2022 | 0.31 |
| 24.60 | 0.95 | 9.12 | 2.69 | 2022 | 0.31 |
| 25.35 | 1.83 | 9.51 | 2.72 | 2022 | 0.31 |
| 25.01 | 1.89 | 9.50 | 2.87 | 2022 | 0.31 |
| 24.91 | 2.11 | 9.48 | 2.84 | 2022 | 0.31 |
| 24.18 | 1.29 | 9.29 | 2.13 | 2022 | 0.31 |

The list math formula to calculate error predicted to the monitoring data measured at the field [2], [3].

1. Mean absolute error (*MAE*):

$$MAE=\frac{1}{n}\sum_{i=1}^{n} \left| y_{i}-\left. \hat{y_{i}} \right| \right.$$

2. Root means square error *(RMSE*):

$$RMSE=\sqrt{\frac{1}{n} \sum_{i=1}^{n} \left( y_{i}-\hat{y_{i}} \right)^{2}}$$

3. Normalized root means square error (*NRMSE* (%)):

$$NRMSE=100\times\frac{RMSE}{ŷ_{max}-ŷ_{min}}$$

4. Goodness-of-fit or coefficient of efficiency R-square (*R^2^*):

$$R^{2}=1-\frac{\sum_{i=1}^{n} \left( y_{i}-\hat{y_{i}} \right)^{2}}{\sum_{i=1}^{n} {(ŷ_{i}-ŷ_{ave})}^{2}}$$

Where:  *y_i_*: predicted values at a time

*ŷ_i_:* the observed values at a time

*ŷ_max_*: the maximum value of observed data

*ŷ_mix_*: the minimum value of observed data

*ŷ_ave_*: the average value of observed data

*n*: number of observations

# Supplementary S2

Table S4. General characteristics of MD, A-0, A-1, and A-2 water chemistry.

|  | Unit | MD | A-0 | A-1 | A-2 |
| --- | --- | --- | --- | --- | --- |
| pH |  | 6.72 | 6.78 | 7.12 | 7.04 |
| OPR | mV | 138 | 178 | 161 | 177 |
| DO | mg/L | 5.02 | 7.33 | 9.18 | 9.48 |
| EC | µS/cm | 1135 | 1138 | 1101 | 1096 |

# Supplementary S3


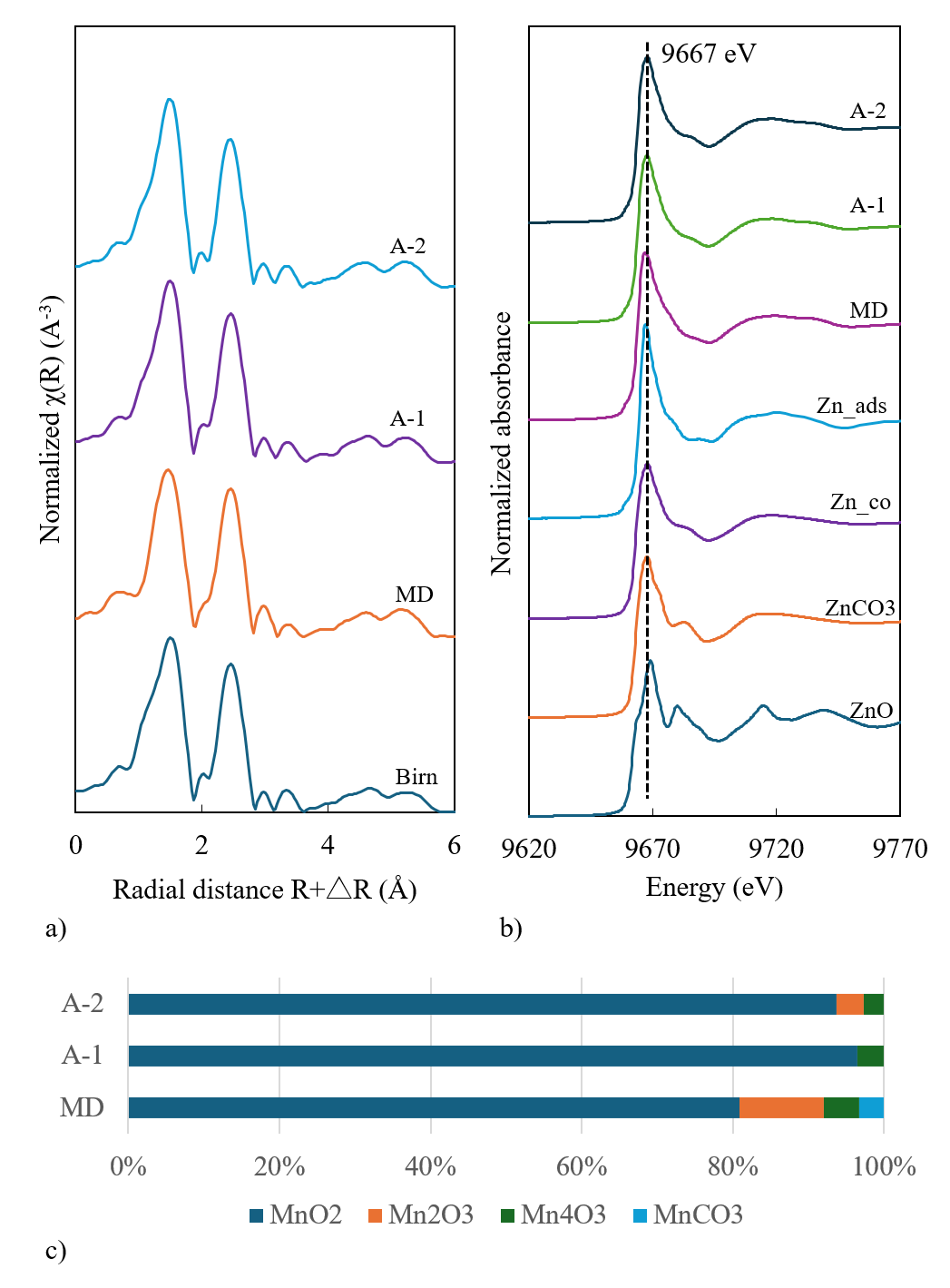


Fig. S1. a) Radal distribution function obtained from Fourier transforms of Mn K-edge EXAFS spectra of the reference material and solid samples; b). Zn K-edge XANES of the reference material and solid samples; Zn_ads: Zn adsorption, Zn coprecipitation; c) The linear fitting combination (LCF) of Mn K-edge XANES energy spectra to quantify the minerals composition in solid samples using XANES spectra energy.

# Supplementary S4

Table S5. The results of XRF analysis of the solid samples

|  | **MDO** | **A-1** | **A-2** |
| --- | --- | --- | --- |
| Oxides | Weight (%) | Weight (%) | Weight (%) |
| LOI-Flux | 20.21 | 35.79 | 16.00 |
| MgO | 0.09 | 0.19 | 0.42 |
| Al_2_O_3_ | 0.07 | 0.07 | 0.13 |
| SiO_2_ | 2.17 | 0.60 | 0.44 |
| P_2_O_5_ | 0.01 | 0.00 | 0.01 |
| SO_3_ | 0.48 | 0.14 | 0.21 |
| Cl | 0.01 | 0.00 | 0.00 |
| K_2_O | 0.28 | 0.07 | 0.09 |
| CaO | 1.25 | 2.15 | 4.10 |
| TiO_2_ | 0.00 | 0.02 | 0.01 |
| Cr_2_O_3_ | 0.00 | 0.01 | 0.01 |
| MnO | 57.94 | 48.23 | 61.38 |
| Fe_2_O_3_ | 9.68 | 1.34 | 0.33 |
| Co_2_O_3_ | 0.03 | 0.02 | 0.02 |
| NiO | 0.00 | 0.01 | 0.01 |
| CuO | 0.12 | 0.02 | 0.02 |
| ZnO | 7.56 | 11.25 | 16.74 |
| As_2_O_3_ | 0.00 | 0.01 | 0.01 |
| SrO | 0.00 | 0.01 | 0.01 |
| Y_2_O_3_ | 0.01 | 0.00 | 0.00 |
| MoO_3_ | 0.01 | 0.01 | 0.01 |
| Cs2O | 0.00 | 0.00 | 0.01 |
| BaO | 0.02 | 0.06 | 0.06 |
| PbO | 0.03 | 0.00 | 0.00 |
| **Total** | **99.99** | **100** | **100** |

Table S6. The results of sequential extraction solid samples by BCR (Community Bureau of Reference) [1]

|  | **Mn (%)** | | | **Zn (%)** | | | **Fe (%)** | | | **Ca (%)** | | |
| --- | --- | --- | --- | --- | --- | --- | --- | --- | --- | --- | --- | --- |
|  | F1 | F2 | F3 | F1 | F2 | F3 | F1 | F2 | F3 | F1 | F2 | F3 |
| MD | 0.08 | 99.79 | 0.14 | 8.13 | 82.30 | 9.57 | 0.00 | 3.51 | 96.49 | 67.62 | 32.38 | - |
| A-1 | 0.03 | 99.92 | 0.05 | 4.92 | 94.22 | 0.86 | 0.00 | 30.23 | 69.77 | 88.74 | 8.49 | 3.07 |
| A-2 | 3.57 | 96.43 | - | 25.00 | 75.00 | - | 0.00 | 93.62 | 6.38 | 98.69 | 1.04 | - |

# Supplementary S5

Table S7. The saturation index (SI) of minerals that possibly form in the pilot-scale passive treatment.

| **Phase** | **SI** | **Log IAP** | **Log K** | **Formula** |
| --- | --- | --- | --- | --- |
| Hausmannite | 11.63 | 72.66 | 61.03 | Mn3O4 |
| Bixbyite | 11.09 | 10.48 | -0.61 | Mn2O3 |
| Pyrolusite | 8.96 | 50.34 | 41.38 | MnO2 |
| Nsutite | 7.78 | 50.34 | 42.56 | MnO2 |
| Birnessite | 6.74 | 50.34 | 43.6 | MnO2 |
| Manganite | 5.41 | 30.75 | 25.34 | MnOOH |
| Rhodochrosite | 1.65 | -9.48 | -11.13 | MnCO3 |
| Rhodochrosite(d) | 0.91 | -9.48 | -10.39 | MnCO3 |
| ZnCO3:H2O | 0.28 | -9.98 | -10.26 | ZnCO3:H2O |
| Calcite | 0.1 | -8.38 | -8.48 | CaCO3 |
| Smithsonite | 0.02 | -9.98 | -10 | ZnCO3 |
| Aragonite | -0.04 | -8.38 | -8.34 | CaCO3 |
| Zincite(c) | -0.48 | 10.66 | 11.14 | ZnO |
| ZnO(a) | -0.65 | 10.66 | 11.31 | ZnO |
| Gypsum | -0.8 | -5.39 | -4.58 | CaSO4:2H2O |
| Zn(OH)2-e | -0.84 | 10.66 | 11.5 | Zn(OH)2 |
| Anhydrite | -1.02 | -5.39 | -4.36 | CaSO4 |
| Zn(OH)2-g | -1.05 | 10.66 | 11.71 | Zn(OH)2 |
| Zn(OH)2-b | -1.09 | 10.66 | 11.75 | Zn(OH)2 |
| Dolomite | -1.28 | -18.37 | -17.09 | CaMg(CO3)2 |
| CO2(g) | -2.49 | -3.95 | -1.47 | CO2 |
| Zn4(OH)6SO4 | -3.4 | 25 | 28.4 | Zn4(OH)6SO4 |
| Zn2(OH)2SO4 | -3.82 | 3.68 | 7.5 | Zn2(OH)2SO4 |
| Pyrochroite | -4.04 | 11.16 | 15.2 | Mn(OH)2 |
| Zn5(OH)8Cl2 | -6.33 | 32.17 | 38.5 | Zn5(OH)8Cl2 |
| ZnSO4:H2O | -6.41 | -6.98 | -0.57 | ZnSO4:H2O |
| MnSO4 | -9.16 | -6.49 | 2.67 | MnSO4 |
| Zincosite | -9.99 | -6.98 | 3.01 | ZnSO4 |
| Zn3O(SO4)2 | -22.33 | -3.31 | 19.02 | ZnO:2ZnSO4 |
| Mn2(SO4)3 | -36.74 | -42.45 | -5.71 | Mn2(SO4)3 |
| ZnMetal | -54.28 | -28.53 | 25.76 | Zn |
| Sulfur | -99.39 | -114.42 | -15.03 | S |
| Sphalerite | -118.41 | -130.03 | -11.62 | ZnS |

Fig. S2. The solubility of field Mn species in water systems obtained from Act2 module of Geochemical Workbench’s

# Supplementary S6

The supplementary S6 is attached in a separate excel file.

# Supplementary S7

Table S8. Summary of the mean (mg/L), standard deviation (STD) (mg/L), and root mean square error (RMSE) (mg/L) for the obtained parameters k_1_=1.03×10^-4^ s_­_^-1^, k_m_=4.11×10^4^ s^-1^, K_d_=4.49 L/g. The predicted model to the monitoring data; Mn_R^2^ = 0.74, Zn_R^2^ = 0.80. Abbreviation: HTR: hydraulic retention time; STD: standard deviation; MAE: mean absolute error; RMSE: root mean square error; NRMSE: normalized root means square error.

|  |  | **Mn^2+^** |  |  |  |  | **Zn^2+^** |  |  |  |
| --- | --- | --- | --- | --- | --- | --- | --- | --- | --- | --- |
| **HTR** | **Mean** | **STD** | **MAE** | **RMSE** | **NRMSE** | **Mean** | **STD** | **MAE** | **RMSE** | **NRMSE** |
| Day | mg/L | mg/L | mg/L | mg/L | % | mg/L | mg/L | mg/L | mg/L | % |
| 2 | 0.20 | 0.22 | 0.20 | 0.30 | 10.9% | 0.57 | 0.35 | 0.34 | 0.49 | 16.5% |
| 0.5 | 0.25 | 0.14 | 0.12 | 0.14 | 4.9% | 1.24 | 0.67 | 0.19 | 0.27 | 9.0% |
| 0.3 | 1.66 | 0.66 | 0.56 | 0.65 | 23.9% | 2.76 | 0.47 | 0.43 | 0.49 | 16.6% |
| Overall | 0.60 | 0.73 | 0.24 | 0.37 | 13.4% | 1.54 | 0.91 | 0.27 | 0.38 | 12.6% |

# References

[1] M. Pueyo, J. Mateu, A. Rigol, M. Vidal, J. F. López-Sánchez, and G. Rauret, “Use of the modified BCR three-step sequential extraction procedure for the study of trace element dynamics in contaminated soils,” *Environmental Pollution*, vol. 152, no. 2, pp. 330–341, Mar. 2008, doi: 10.1016/j.envpol.2007.06.020.

[2] N. (Klaas) M. Faber, “Estimating the uncertainty in estimates of root mean square error of prediction: application to determining the size of an adequate test set in multivariate calibration,” *Chemometrics and Intelligent Laboratory Systems*, vol. 49, no. 1, pp. 79–89, Sep. 1999, doi: 10.1016/S0169-7439(99)00027-1.

[3] D. R. Legates and G. J. McCabe, “Evaluating the use of ‘goodness‐of‐fit’ Measures in hydrologic and hydroclimatic model validation,” *Water Resources Research*, vol. 35, no. 1, pp. 233–241, Jan. 1999, doi: 10.1029/1998WR900018.
